# Behavioral responses of Atlantic cod to sea temperature changes

**DOI:** 10.1002/ece3.1496

**Published:** 2015-04-17

**Authors:** Carla Freitas, Esben Moland Olsen, Even Moland, Lorenzo Ciannelli, Halvor Knutsen

**Affiliations:** 1 Department of Natural Sciences, Faculty of Engineering and Science, University of AgderPost Box 422, 4604, Kristiansand, Norway; 2 Institute of Marine ResearchFlødevigen, 4817, His, Norway; 3 Centre of Marine and Environmental Research (CIIMAR/CIMAR), University of Porto4050-123, Porto, Portugal; 4 Department of Biosciences, Centre for Ecological and Evolutionary Syntheses (CEES), University of OsloPO Box 1066, Blindern, 0316, Oslo, Norway; 5 College of Earth, Ocean, and Atmospheric Sciences, Oregon State University104 CEOAS Administration Building, Corvallis, Oregon, 97331-5503

**Keywords:** Acoustic telemetry, climate change, diel vertical migration, *Gadus morhua*, sea surface temperature

## Abstract

Understanding responses of marine species to temperature variability is essential to predict impacts of future climate change in the oceans. Most ectotherms are expected to adjust their behavior to avoid extreme temperatures and minimize acute changes in body temperature. However, measuring such behavioral plasticity in the wild is challenging. Combining 4 years of telemetry-derived behavioral data on juvenile and adult (30–80 cm) Atlantic cod (*Gadus morhua*), and in situ ocean temperature measurements, we found a significant effect of sea temperature on cod depth use and activity level in coastal Skagerrak. During summer, cod were found in deeper waters when sea surface temperature increased. Further, this effect of temperature was stronger on larger cod. Diel vertical migration, which consists in a nighttime rise to shallow feeding habitats, was stronger among smaller cod. As surface temperature increased beyond ∼15°C, their vertical migration was limited to deeper waters. In addition to larger diel vertical migrations, smaller cod were more active and travelled larger distances compared to larger specimens. Cold temperatures during winter tended, however, to reduce the magnitude of diel vertical migrations, as well as the activity level and distance moved by those smaller individuals. Our findings suggest that future and ongoing rises in sea surface temperature may increasingly deprive cod in this region from shallow feeding areas during summer, which may be detrimental for local populations of the species.

## Introduction

Considerable changes in climate are expected in the near future (IPCC 2013). Sea surface temperatures are expected to rise, with increases over 3°C being predicted in some areas of the North Atlantic by the end of this century (Sheppard 2004; Dye et al. 2013). It is therefore essential to understand how wild marine fish respond to changes in their ambient temperature.

Fish are ectotherms, meaning that their body temperatures conform to surrounding water temperatures. Experiments demonstrate that fish physiology and behavior are highly affected by water temperature (see Fry 1971; Olla et al. 1978; Pörtner and Farrell 2008). Increases in temperature beyond their natural temperature range (their so-called thermal window) limit the capacity of circulatory and ventilatory systems to match oxygen demands, resulting in a decrease in the animal's capacity to perform aerobically (Brett 1971; Nilsson et al. 2009; Eliason et al. 2011). This decline in aerobic scope affects critical biological functions, including growth, reproduction, muscular activity, and behavior (see Pörtner and Knust 2007; Pörtner and Farrell 2008). Further temperature increases lead to growth cessation, anaerobic respiration, protein denaturation, permanent inactivation of enzymes, and eventually death (Katersky and Carter 2007; Pörtner and Knust 2007; Wang and Overgaard 2007). Ectotherms in the wild usually make some behavioral attempt (so-called thermoregulatory behavior) to avoid extreme temperatures and minimize acute changes in body temperature (McCue 2004). Fish may also respond to temperature changes by acclimatization, which involves compensatory shifts in physiological parameters (Alvarez et al. 2004; Grenchik et al. 2013). However, poleward shifts in the distribution of various fish species (Perry et al. 2005; Poloczanska et al. 2012; Engelhard et al. 2014) indicate a response consistent with a limited ability by these species to adjust their thermal window to warming ocean temperatures.

**Figure 1:**
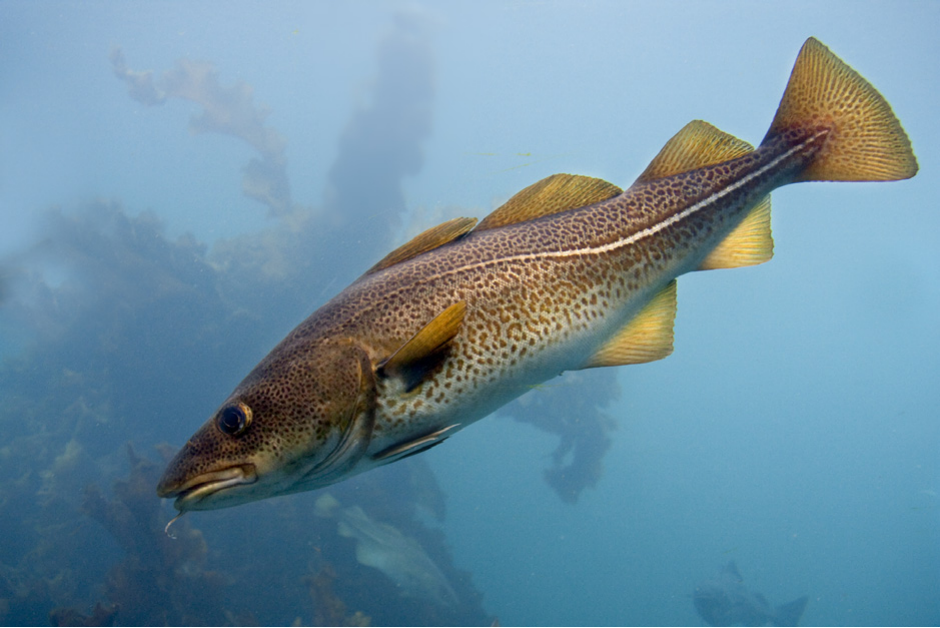
Atlantic cod (*Gadus morhua*) in coastal Skagerrak (photograph by Øystein Paulsen).

Increases in sea temperatures have given rise to concern on some populations of Atlantic cod *Gadus morhua* (Fig.1; Drinkwater 2005; Mieszkowska et al. 2009). The cod is found demersally throughout the North Atlantic and holds a major economical and ecological importance (Worm and Myers 2003; Frank et al. 2005). Several stocks of the species have declined dramatically in the last decades due to overfishing (Horwood et al. 2006; Hutchings and Rangeley 2011). Climate is also thought to exert a strong control on cod stocks, both directly through changes in distribution and recruitment (Perry et al. 2005; Stige et al. 2006; Engelhard et al. 2014) and indirectly by its influence on plankton prey for cod larvae (Beaugrand and Kirby 2010; Johannessen et al. 2011). The range of temperatures that cod occupies in natural conditions has been investigated using combined hydrographic and fishing surveys (Blanchard et al. 2005; Rindorf and Lewy 2006), as well as directly using data-storage tags (Pálsson and Thorsteinsson 2003; Neat and Righton 2007; Righton et al. 2010). While the response of cod to temperature has been studied in several laboratory-based studies (see Metcalfe et al. 2012 and references therein), measuring responses in the wild is challenging as it requires fish behavior and sea temperature data at the same spatial and temporal scales.

Using 4 years of telemetry-derived behavioral data and in situ daily temperature measurements, we investigated whether sea temperature affected the behavior of wild-ranging cod at the Norwegian Skagerrak coast. The effect of other physical parameters (e.g., precipitation and upwelling events) on cod behavior was also investigated, as well as the role of fish body length in shaping responses to these drivers. Body length is a key phenotypic trait that can affect temperature preference (Lafrance et al. 2005) and individual behavior (e.g., diel vertical movements; Olsen et al. 2012).

## Materials and Methods

### Biotelemetry

**Figure 2:**
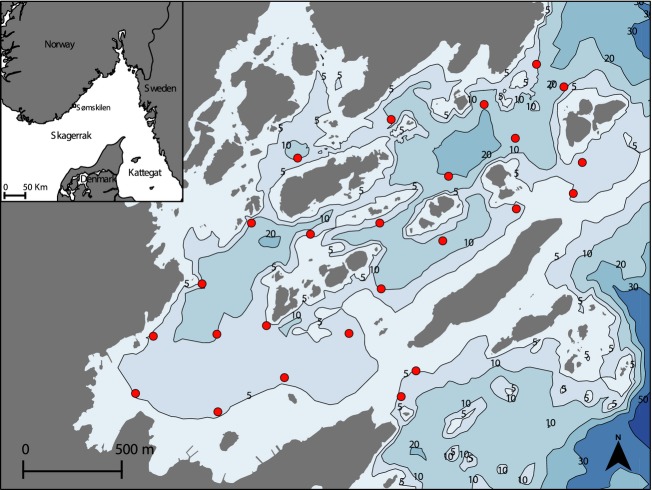
Map of the study area (Sømskilen), showing the depth contours and the network of 25 acoustic receivers (red dots) used to monitor tagged Atlantic cod.

The biotelemetry study was conducted in Sømskilen bay, located in the central part of the Norwegian Skagerrak coast (Fig.2). Sømskilen is a shallow, semisheltered basin with numerous small islands and skerries. The upper 5–7 m are composed of rocky bottoms covered by macroalgae or sandy bottoms with eelgrass (Espeland et al. 2010). The deepest areas (5–40 m) consist mainly of mud flats with sparse vegetation (Espeland et al. 2010). Three channels allow fish to move to and from the bay. Sømskilen is influenced by the outflow of the river Nidelva, which discharges via two channels: one inside our study area and the other just outside on the eastern side (Fig.2). Its freshwater discharge into Sømskilen is variable, but limited to the surface layer (Espeland et al. 2010). In coastal Skagerrak, cod forms a network of local populations with limited connectivity (Knutsen et al. 2003; Ciannelli et al. 2010) and with an overall decline in abundance in recent decades (Olsen et al. 2009).

Cod were collected in the Sømskilen bay in May 2008, 2011, and 2012, using fyke nets (Table1). Fishes were selected for tagging based on their body length and position of capture within the study area, as we aimed to tag roughly the same number of fishes throughout the available range of body sizes and study area. All cod were measured to the nearest cm (fork length) and brought either 3 km to the Flødevigen Research Station (Institute of Marine Research) or to the nearest shore for tagging. A total of 191 cod, ranging in size from 30 to 80 cm, were tagged (Table1).

**Table 1:** Summary statistics of 181 Atlantic cod used in the study. Individuals not tracked beyond the tagging month (*N* - 10) were excluded from the analyses and from this table

Tagging year	*N* individuals	Body length (cm)		Data range (days)		*N* detections	*N* depth records
		Mean	Range	Mean	Range		
2008	60	44.9	30–66	120	5–395	1,534,449	632,143
2011	49	45.9	31–69	177	18–500	3,980,195	1,269,729
2012	72	47.7	30–80	115	17–196	2,935,733	977,829
Total	181	46.3	30–80	134	5–500	8,450,377	2,879,701

Before tagging, the cod were anesthetized with clove oil. Then an ultrasonic transmitter (Vemco V9P-2L, 38 × 9 mm, weight in seawater <3 g) was surgically implanted through a small incision on the ventral surface of the peritoneal cavity. The incision was closed with two absorbable sutures. All cod were also tagged with an external anchor T-bar tag (Hallprint TBA1, 30 × 2 mm) printed with a serial number and a reward notice. Fishes were then released at the site of capture. In the first year (2008), fishes were kept and observed in a large aquarium for 1–3 days before releasing. There was no tagging mortality and all fishes looked healthy and immediately swam toward the bottom when released.

The transmitters were built with a pressure sensor (0.44 m resolution, 5 m accuracy and 100 m maximum depth) and programmed to emit a unique identification code at random intervals every 110–250 sec. They were also programmed to transmit the current depth in each second transmission in 2008 and in every transmission in the subsequent years. Expected battery lifetime was 508–660 days.

Underwater ultrasonic receivers (Vemco VR2W, 69 kHz) were used to record signals emitted by the fish transmitters. A total of 25 receivers were placed in the study area in May 2008 (Fig.2). Receivers were positioned to ensure comprehensive monitoring of the study area. This was confirmed using range tests (Olsen and Moland 2011). The same range tests showed that most transmissions within 400 m were detected by the receivers (Fig. S1). Receivers logged the exact time, date, depth, and transmitter code of a given emission. Recorded data were periodically downloaded from the receivers. Data used in this study were collected up to December 2012 (Table1). Note that some cod tagged in 2008 were still sending data in 2009 (see Table1). Therefore, our dataset included fish data from 2008, 2009, 2011, and 2012.

### Hydrographic variables

**Figure 3:**
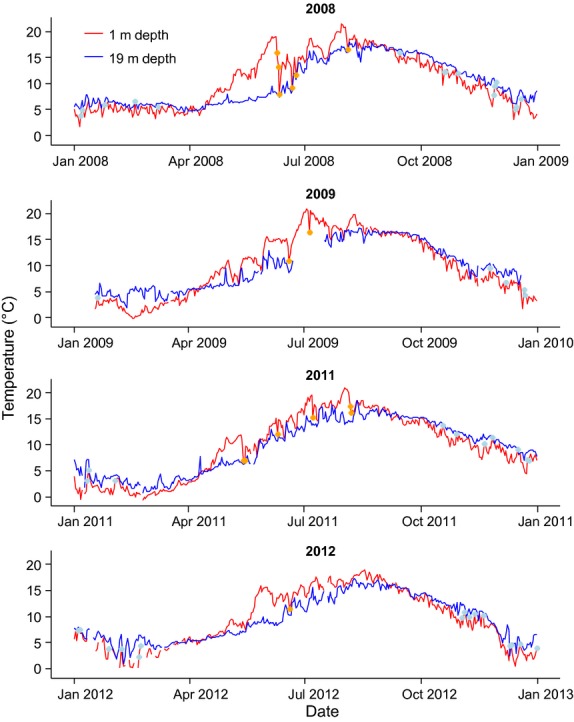
Surface (1 m depth) and bottom (19 m depth) temperatures registered in Flødevigen at 08:00 GMT in 2008, 2009, 2011, and 2012. Upwelling days (identified by surface temperature drops larger than 3°C in summer or surface temperature rises larger than 2°C in winter; see Materials and Methods) are marked with orange and blue dots in summer and winter, respectively.

Water temperatures at 1 and 19 m depth, collected daily at 08:00 UTC at the Flødevigen Research Station, were used in this study. Temperatures were measured using an Aanderaa 4120 sensor with 0.1°C accuracy. Sea temperatures measured at Flødevigen are highly correlated with records from elsewhere in the Skagerrak (Rogers et al. 2011) and therefore likely representative of temperature changes in Sømskilen, located 3 km away. Temperatures at 1 and 19 m are hereafter referred to as surface and bottom temperatures, respectively. Surface temperatures were in general warmer than bottom temperatures in summer, with differences reaching >5°C on several occasions (Fig.3). Such periods of thermal stratification were interrupted on some occasions by sudden decreases in surface temperatures, due to upwelling or turbulent-mixing events. In winter, surface temperatures were in general colder than bottom temperatures, with upwelling/mixing events leading to sudden increases in surface temperatures (Fig.3). Given this dissimilarity in hydrographic conditions, two seasons were defined in this study: summer and winter. Summer extended from the day when surface temperature started to exceed bottom temperatures (sometime in the beginning of April), up to end of August or beginning of September when surface temperatures decreased again below bottom temperatures; winter was defined as the complementary period (see Fig.3).

Differences in surface temperature between consecutive days were used in this study to infer the presence of upwelling/mixing events (hereafter referred to as upwelling events). An upwelling event was considered to have occurred when surface temperatures dropped 3 or more °C in 24–48 h (summer) or increased 2 or more °C in 24–48 h (winter; Fig.3).

Precipitation was used in this study as a proxy for surface salinity and suspended sediments, under the hypothesis that large precipitation events and consequent increases in the river inflow would decrease surface salinities and increase water turbidity. Daily precipitation (amount of precipitation in the last 24 h, recorded at 06:00 UTC) was obtained from the Norwegian Meteorological Institute station Bøylefoss, located 20 km upstream on the river Nidelva.

### Data processing

Ultrasonic receiver data were exported from Vemco VUE software (version 2.0.5) into R software (R Core Team 2012) where all analyses were performed. Receiver clocks may drift over time and thus loose or gain time (Vemco 2012). Data were therefore corrected for temporal drift using a linear correction, assuming that time drifts linearly with time and that laptop times were correct when the receivers were initialized and when data were downloaded.

Any isolated detection occurring in a 24-h period was removed as potentially spurious. The tagging month was also removed, so that all individual datasets from the same year started at the same day and potential post-tagging effects on behavior (normally visible in the dataset during the first hours) minimized. Redundant depth observations (recorded at the same time by more than one receiver) were removed (except when estimating center-of-activity locations; see below). Depth recordings were transmitted twice as often in 2011 and 2012 compared to 2008 due to the transmission protocol described above. In order to make all depth data comparable, we resampled the 2011 and 2012 data (after repeated observations had been removed) so that only each second depth was retained.

The following behavior metrics were obtained for each fish for each day: (1) average depth during the day; (2) average depth during the night; (3) diel vertical migration range; (4) activity level, that is, average short-term changes in depth; and (5) average distance moved per hour.

For a given day, average depth during the day was calculated as the average of all depths recorded between sunrise and sunset (i.e., when solar elevation was ≥0). Average depth during the night was calculated as the average of all depths recorded from sunset in the previous day to sunrise of that day. Diel vertical migration range was calculated as the difference between average depth during the day and average depth during the night. Average short-term changes in depth were used here as a proxy for the fish activity level. In order to estimate this metric, the standard deviation in depth for every 1-h period was calculated and then averaged for each given day. Finally, average distance moved per h was estimated from center-of-activity locations (Simpfendorfer et al. 2002). The center-of-activity location for a given time interval *t* is the mean position of the receivers that detected the animal at that time interval, weighted by the number of times the animal was detected at each receiver (see Simpfendorfer et al. 2002). When an animal is detected by a single receiver during time *t*, then the center-of-activity location will be at the exact location of the receiver. A small *t* increases the probability of centers-of-activity being estimated from single receivers, resulting therefore in few unique center-of-activity locations. Large *t* values on the other hand also result in few center-of-activity locations due to the reduced temporal resolution. We found that, in the present study, a *t* between 30 and 60 min maximized the number of unique center-of-activity locations (Fig. S2) and decided to use the upper limit (*t* - 60 min). The distance between consecutive center-of-activity locations was calculated and then divided by the number of hours between them (as detections were absent in some 60-min intervals). Distances between locations more than 24 h apart were excluded. A maximum of 24 distances were obtained for each day, and these were then averaged to obtain the average distance moved per h in that day.

### Data modeling

Before applying any statistical models, data exploration was carried out following the protocol described in Zuur et al. (2010). Linear mixed-effects models were used to investigate the effect of hydrographic conditions and fish body size (explanatory variables) on fish behavior (response variable). Five models were independently developed, one for each behavior metric. Because hydrographic conditions differed greatly in summer and winter (see above; Fig.3), models were fitted for summer and winter separately (totalizing therefore 10 independent models). Models were fitted using the R package *nlme*. The following explanatory variables were used in the models: T1 m (surface temperature, in °C), T19 m (bottom temperature, in °C), DifT (difference between surface and bottom temperature, in °C), Up (categorical variable indicating whether upwelling was present or not; see above), Prec (precipitation, in mm), and Len (body length, in cm). In addition, individual identity and year were considered as random effect variables.

Models were selected by first looking for the optimal random structure and then for the optimal fixed structure (Zuur et al. 2009). We started with a model containing the full suite of fixed effects and searched for the optimal random error structure using the Akaike information criteria, AIC (Burnham and Anderson 2002), with restricted maximum likelihood (REML) estimation procedure. Individual identity, nested within year gave the best fit (Tables S1–S10). We hypothesized that both the intercept and the slope of the effect of sea temperature on cod behavior could change from individual to individual. We therefore included random slopes in the random structure in addition to random intercepts. Autocorrelation functions showed temporal autocorrelation of model residuals. An autoregressive term was therefore added to the models to account for the nonindependence of the error (Dormann et al. 2007). Using the AIC as a model selection tool, the autoregressive process of order 1 (implemented with the corAR1 function in R) was selected (Tables S1–S10). Once the optimal random component structure was found, the optimal structure of the fixed effects was explored using forward selection based on the AIC, with maximum likelihood (ML) parameter estimation. Surface and bottom temperatures were highly correlated (Pearson correlation coefficients *r* - 0.7 in summer; *r* - 1.0 in winter). They were thus not used simultaneously in the models to avoid collinearity and related problems with parameter estimations (Zuur et al. 2009). We excluded the one explaining less deviance by comparing the AIC of models with only one predictor and no random slopes (Tables S1–S10). Variables Up and DifT were not used simultaneously in the models, as these were related (upwelling events resulted in none or small differences between surface and bottom temperatures). We hypothesized that upwelling events could lead cod to change their vertical position in the water column and therefore used this variable when fitting the models on the average depth used at day and night. We also hypothesized that the range of the diel vertical movements could be affected by the differences in temperature between surface and bottom waters and therefore used the covariate DifT when predicting diel vertical migration ranges. Finally, we hypothesized that both upwelling events and differences in temperatures could potentially affect the activity level and distance moved and therefore used the covariate explaining the greatest variance (Tables S1–S10). All other pairs of variables were not highly correlated (*r* ≤ 0.5). In addition to the main effects of the explanatory variables, the following interactions were considered: T1 m x Len (to test whether the effect of summer surface temperatures on fish behavior was stronger or weaker depending on fish body size) and Up x T1 m (in order to verify whether the effect of upwelling events was weakened or strengthened when summer surface temperatures increased). Continuous variables with no true zero, used in the interactions (T1 m and Len), were previously centered, by subtracting the sample mean (15.8°C and 46.3 cm, respectively) to each observation. Centering of these variables ensured a biologically meaningful interpretation of the model estimates.

Coefficients from the resulting optimal models were estimated using restricted maximum likelihood (REML). These models were validated following Zuur et al. (2009) to verify that the underlying statistical assumptions were not violated. Average short-term changes in depth and average distance moved per hour had to be square-root transformed in order to reach homogeneity of variances and normality of model residuals.

When the model with lowest AIC value has an Akaike weight value lower than 0.9, a model averaging procedure might be more appropriate to account for parameter uncertainty (Burnham and Anderson 2002). Therefore, we constructed a 95% confidence set of models where the sum of Akaike weights was >0.95, following Louzao et al. (2009). Accordingly, averaged coefficients were estimated from the 95% confidence set of models containing that variable, as well as a variance estimator in order to assess the precision of the estimates (Burnham and Anderson 2002).

Observed data and model residuals indicated some nonlinearities in the relationship between cod behavior (average depth) and temperature. In order to better model such nonlinearities generalized additive mixed models, GAMMs (Wood 2006) were fitted using the same random and fixed-effect structure as selected by the linear models. We used a Gaussian distribution and identity link. We fitted the models using the R package *mgcv*.

## Results

In total, vertical and horizontal movements of 181 cod (Table1) were monitored during 2008–2012. From this, over 8 million acoustic detections and 2.8 million depth recordings were obtained (Table1). Cod were detected in depths ranging from 0 to 40 m in summer and 0–34 m in winter.

**Figure 4:**
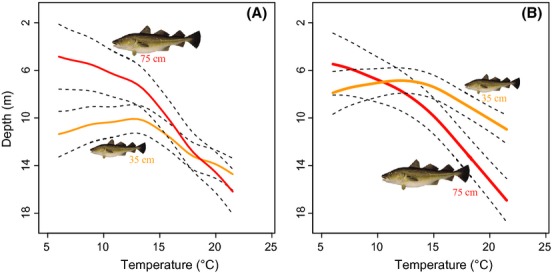
Average depth used by cod during the day (A) and the night (B) as a function of sea surface temperature, as predicted from generalized additive mixed models (GAMMs). Predictions are given for a 35 cm (orange lines) and 75 cm cod (red lines) during summer. Solid lines are estimated mean effects and dashed lines are 95% pointwise confidence intervals.

**Figure 5:**
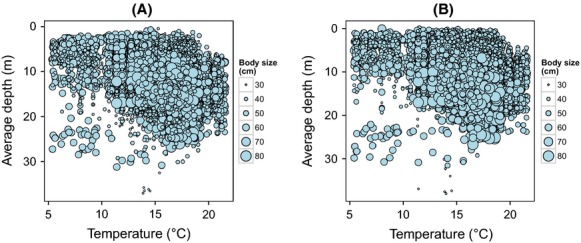
Average depth used by cod during the day (A) and night (B) as a function of sea surface temperature during summer. Each point refers to a fish in a given day. Symbol size is relative to fish body size. Note the absence of cod, especially larger individuals, from the first meters of the water column at increased sea surface temperatures (upper right corner).

Linear mixed models and generalized additive mixed models, accounting for temporal autocorrelation and for random variability between individuals, showed a significant effect of sea surface temperature on the vertical position occupied by cod during summer. Depth used by cod increased significantly as surface temperature increased, both during daytime and nighttime (Table2, Fig4). Further, this effect of temperature was stronger on larger fish, as indicated by the positive interaction between surface temperature and fish body size (Table2; Fig.4). The observed data show the absence of cod, especially larger individuals, from the upper meters of the water column at increased sea surface temperatures (Fig.5).

**Table 2:** Summary of linear mixed-effects models used to explain the behavior (response) of cod during summer. Averaged parameter estimates (*β*) are given together with the corresponding standard errors (SE), degrees of freedom (df), 95% confident intervals (95% CI), and number of models within the 95% confidence set (*N* models). Bold values indicate significant effects (95% CI that do not include zero)

Response	Parameter	*β*	SE	df	95% CI		*N* models
AvDepthDay	T1 m	**0.620**	0.049	14,602	**0.525**	**0.715**	1
	Up	−0.037	0.156	14,602	−0.342	0.268	
	Len	−0.036	0.022	193	−0.079	0.006	
	Up x T1 m	**−0.355**	0.038	14,602	**−0.429**	**−0.281**	
	T1 m x Len	**0.012**	0.003	14,602	**0.006**	**0.018**	
AvDepthNight	T1 m	**0.510**	0.031	14,430	**0.448**	**0.572**	1
	Up	**−0.713**	0.157	14,430	**−1.020**	**−0.406**	
	Len	**0.072**	0.022	193	**0.029**	**0.115**	
	Up x T1 m	**−0.354**	0.038	14,430	**−0.428**	**−0.280**	
	T1 m x Len	**0.013**	0.003	14,430	**0.007**	**0.019**	
DVM	DifT	**−0.070**	0.020	14,292	**−0.109**	**−0.031**	2
	T19 m	**0.125**	0.016	14,292	**0.094**	**0.157**	
	Len	**−0.107**	0.018	193	**−0.142**	**−0.072**	
	Prec	−0.004	0.003	14,292	−0.009	0.002	
Activity	Up	0.009	0.010	14,422	−0.010	0.027	4
	T19 m	**−0.005**	0.001	14,422	**−0.007**	**−0.004**	
	Len	**−0.003**	0.001	193	**−0.004**	**−0.001**	
	Prec	0.000	0.000	14,422	−0.001	0.000	
Dist	T19 m	**0.085**	0.013	14,582	**0.059**	**0.111**	3
	DifT	**0.090**	0.015	14,582	**0.060**	**0.120**	
	Prec	0.004	0.002	14,582	0.000	0.008	
	Len	**−0.034**	0.013	193	**−0.061**	**−0.008**	

AvDepthDay, average depth during the day (m); AvDepthNight, average depth during the night (m); DVM, diel vertical migration (m); Activity, short-term standard deviation in depth (m; sqrt transformed); Dist, average distance moved per h (m; sqrt transformed); T1 m, temperature at 1 m depth, that is, surface temperature (°C); T19 m, temperature at 19 m depth, that is, bottom temperature (°C); DifT, difference between surface and bottom temperatures (°C); Up, upwelling (two categories: 0 - absence, 1 - presence); Prec, precipitation (mm), Len, fish body size (cm). Covariates T1 m and Len were centered by subtracting the mean before inclusion in the models that included interactions.

**Figure 6:**
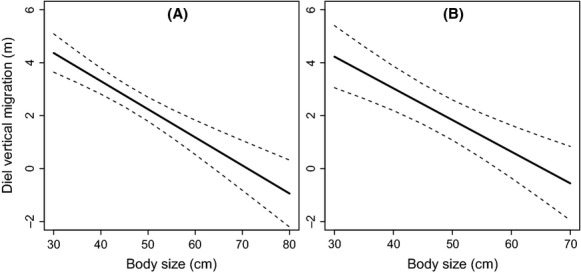
Diel vertical migration (difference between average depth during the day and average depth during the night), as a function of cod body size, as predicted from generalized additive mixed models (GAMMs) for summer (A) and winter (B). Solid lines are estimated mean effects and dashed lines are 95% pointwise confidence intervals.

Our results show that larger cod tended to use similar depths during day and night (Fig.4). Smaller individuals, on the other hand, stayed deeper during the day but rose to shallower waters at night (Fig.4). Accordingly, models show that diel vertical migration was stronger among smaller cod (Table2, Fig.6A). Increases in surface temperature, rather than affecting the magnitude of diel vertical migration, tended to shift diel vertical migration toward deeper waters (Fig.4). In addition to displaying larger diel vertical migrations, the smaller individuals in this study were also more active and moved larger distances compared to larger specimens (Table2). We found a slight decrease in the magnitude of diel vertical migration under thermal stratification (Table2). Upwelling, in contrast, triggered the rise of fish to shallow waters at night, as shown by the negative relationship between upwelling and nighttime depth (Table2). Note, however, that the effect of upwelling on cod nighttime depth was weaker when surface temperatures were high (Table2).

A significant effect of sea temperature on fish behavior was also found during the winter. Decreased surface temperatures resulted in the use of deeper (warmer) waters during the night independently of body size (Table3). During the day, however, larger fish tended to stay in shallower (colder) areas compared to smaller ones (Table3). This pattern was in fact also observed during summer when sea surface temperatures were low (see Fig.4). Similar to summer, smaller fish displayed larger diel vertical migrations, were more active, and travelled larger distances (Fig.6B, Table3). Cold surface temperatures tended, however, to reduce the magnitude of diel vertical migrations (Table3), as well as the activity level and distance moved by those smaller individuals (Table3).

**Table 3:** Summary of linear mixed-effects models used to explain the behavior (response) of cod during winter. Averaged parameter estimates (*β*) are given together with the corresponding standard errors (SE), degrees of freedom (df), 95% confident intervals (95% CI), and number of models within the 95% confidence set (*N* models). Bold indicate effects (95% CI that do not include zero)

Response	Parameter	*β*	SE	df	95% CI		*N* models
AvDepthDay	T1 m	0.001	0.030	5391	−0.057	0.059	2
	Up	0.061	0.133	5391	−0.200	0.323	
	Len	**−0.114**	0.044	71	**−0.201**	**−0.027**	
	Prec	−0.004	0.004	5391	−0.011	0.003	
AvDepthNight	T1 m	**−0.097**	0.026	5431	**−0.147**	**−0.046**	7
	Up	−0.146	0.096	5431	−0.334	0.042	
	Len	0.001	0.038	71	−0.074	0.076	
	Prec	−0.003	0.003	5431	−0.008	0.002	
DVM	DifT	0.001	0.049	5335	−0.094	0.097	2
	T1 m	**0.159**	0.029	5335	**0.102**	**0.216**	
	Len	**−0.117**	0.027	71	**−0.170**	**−0.064**	
	Prec	0.001	0.004	5335	−0.007	0.009	
Activity	Up	0.003	0.009	5869	−0.015	0.021	5
	T1 m	**0.005**	0.001	5869	**0.002**	**0.008**	
	Len	**−0.004**	0.001	71	**−0.006**	**−0.001**	
	Prec	0.001	0.000	5491	0.000	0.001	
Dist	T1 m	**0.092**	0.022	5484	**0.049**	**0.136**	4
	DifT	−0.050	0.039	5484	−0.127	0.027	
	Prec	−0.002	0.003	5484	−0.009	0.004	
	Len	−0.042	0.023	71	−0.088	0.004	

See Table2 for abbreviations, except for DifT, which in this case is the difference between bottom and surface temperatures (°C).

## Discussion

A clear association was found between sea surface temperature and the vertical position occupied by cod both during summer and winter. During summer, cod were found in deeper, colder waters when surface temperature increased. We propose that this response to temperature is likely to reflect physiological constraints of this cold-water species. Similarly to other fish species, cod physiology is profoundly affected by environmental temperature (Jobling 1988; Brander 1995; Claireaux et al. 2000; Pörtner et al. 2001; Björnsson and Steinarsson 2002; Lannig et al. 2004; Yoneda and Wright 2005). Deviations in temperature beyond thermal limits induce a progressive mismatch between oxygen supply and oxygen demand, which will, in turn, firstly cause a decrease in whole organism performance and finally become lethal toward extreme temperatures (see Pörtner et al. 2008). Wild-ranging cod can be found in waters ranging from −1.5 to 19°C, although most observations occur in waters below 15°C (Blanchard et al. 2005; Rindorf and Lewy 2006; Righton et al. 2010). Tank studies indicate that cod's thermal preference may range from 3 to 15°C, depending on factors such as fish body size, hemoglobin genotype, and dissolved oxygen concentrations (Petersen and Steffensen 2003; Lafrance et al. 2005; Behrens et al. 2012). Optimal temperatures for growth range between 9 and 15°C depending on fish body size (Jobling 1988; Björnsson and Steinarsson 2002; Björnsson et al. 2007). Growth experiments at 20°C have resulted in not only low growth, but also high mortality rates (Björnsson et al. 2007). Interestingly, cod in this study sharply veered toward deeper waters when temperatures increased above 15°C.

The observed movement of cod toward deeper, colder waters could alternatively be mediated by indirect effects, such as prey distribution. However, cod is generally adapted to benthic feeding (Brawn 1969). In the Norwegian Skagerrak coast, the diet of juvenile and adult cod (>30 cm) is composed of a variety of invertebrates (such as crabs, shrimps, gastropods, and polychaetes) and benthic fish, mostly gobies (Hop et al. 1992). There are no indications that these prey species migrate to deep waters as surface temperatures increase as they can be found in large numbers in shallow nearshore habitats throughout the warm summer months in southern Norway (Johannessen 2014).

In our study, the avoidance of warm water was especially evident in larger cod. Indeed, larger fish may experience problems with oxygen supply at lower temperatures than smaller fish (Pörtner et al. 2008; Pauly 2010). There is also evidence that increasing water temperatures will favor small body sizes (e.g., Daufresne et al. 2009; Baudron et al. 2014). For Atlantic cod in particular, laboratory experiments have shown that temperature preference and optimal temperature for growth decreases with increased body size (Lafrance et al. 2005; Björnsson et al. 2007). The size-dependent response to temperature observed in this study likely reflects the avoidance of temperatures that were stressful or detrimental to growth.

Vertical movements of adult (>56 cm) cod toward colder waters were previously found by Meager et al. (2012) during the spawning season in spring in the western coast of Norway. On a wider scale study, Neat and Righton (2007) monitored the ambient temperature used by juvenile and adult (>30 cm) cod, equipped with data-storage tags, and found that North Sea cod occupied suboptimal areas; that is, they appeared to choose to stay in warmer waters even when colder, hypothetically more suitable temperatures were available at the bottom of the North Sea. They therefore suggest that adult cod do not move toward cold-water masses. The coarse temporal and spatial scales used (bottom temperatures pooled across several years and animal data pooled over wide geographic areas) may have hindered detection of individual and time-resolved responses. Note also that both Meager et al. (2012) and our study report that surface temperature, rather than bottom temperatures, were the best predictors of cod vertical dynamics.

We found that smaller individuals displayed stronger diel vertical migrations, similarly to what was previously reported by Olsen et al. (2012). Diel vertical migrations are cyclic changes in the position of aquatic organisms in the water column that occur with 24-h periodicity (Neilson et al. 1990; Mehner 2012). It is thought to be caused by the trade-offs between feeding opportunities and predator avoidance and/or bioenergetic efficiency (Neilson et al. 1990; Mehner 2012). The significant inverse correlation between diel vertical migration and body size is unlikely to be related to predator avoidance as smaller individuals are more susceptible to predators during their nighttime excursions to shallow waters. Olsen et al. (2012) found that, during summer, fish with strong diel vertical migration had a higher risk of being captured in the fishery. They hypothesized that this pattern could lead to selection against shallow-water excursions, meaning that the surviving bigger fish were those with stationary deep water behavior. Other possible explanations include physiological reasons for the depth distribution as larger cod seem to be more sensitive to warm temperatures as discussed above. Size-dependent differences in diel vertical migrations may result or be a result of changes in diet as fish grow. Hop et al. (1992) reported that the diet of cod in coastal Skagerrak (composed of a diversity of benthic invertebrates and fish) varied both seasonally and with body size. Finally, these differences in diel vertical migration may also reflect ontogenetic changes in energy requirements. Smaller fish generally have higher metabolic rates and stronger demands for surplus energy to maximize growth and energy storage (Brett and Groves 1979; Post and Parkinson 2001). They may therefore spend more time foraging than larger individuals (Martelo et al. 2013). The smaller individuals in this study, in addition to displaying larger diel vertical migration, were also more active and moved larger distances compared to larger specimens, which supports the latter hypothesis.

While thermal stratification in summer tended to decrease the magnitude of diel vertical migration, upwelling, in contrast, triggered the rise of fish to shallow waters at night. It is possible that upwelling events increase feeding opportunities at the surface, in addition to generally leading to decreased surface temperatures. This result suggests that not only seasonal, but also rapid changes in temperature (due to upwelling) drive plastic changes in diel vertical migration, modifying the underlying trade-offs for this behavior and habitat use.

Cod was seen to respond behaviorally to temperature also during winter. Opposite to summer, surface temperatures in winter were colder than bottom temperatures (see Materials and Methods). Decreased surface temperatures resulted in the use of deeper (warmer) waters during the night, independent of body size. During the day, however, larger fish tended to stay in shallower areas than smaller ones. This pattern was also observed during summer when sea surface temperatures were low. This may be related to a higher preference of large fish for colder waters, although the tolerance to temperatures close to zero, in terms of growth, appears to be relatively similar between size classes (Björnsson et al. 2007). Cold temperatures tended to reduce the magnitude of diel vertical migration, as well as activity level and distance moved. These observations are likely related to the fact that low temperatures tend to depress metabolic rates in fish (Johnston and Dunn 1987).

No relationship was found between the amount of precipitation and any of the analyzed behavioral metrics. Large precipitation events are likely to reduce surface salinities and may also increase water turbidity. Because of the presence of reservoirs along the Nidelva River, the fresh water discharge in the study area might not have been proportional to the amount of precipitation. Possible effects of changes in salinity and water turbidity on cod behavior in relation to the distribution of feed organisms require further research.

Results from this study indicate that an ecological consequence of rising temperature is that coastal cod may increasingly abandon shallow-water habitats during warm summer periods. Observed data show the absence of cod, especially larger individuals, from the upper meters of the water column when sea surface temperature increased. Such shallow areas comprise unique habitats, such as eelgrass and macroalgae beds (Espeland et al. 2010), which may become unavailable for cod during warm periods. Although further research is needed to investigate the effect of temperature on cod habitat selection, it is known that eelgrass and macroalgae habitats, available in shallow areas, are highly profitable for cod in terms of energy intake (Persson et al. 2012). In Skagerrak, cod exploit these areas during the night (Espeland et al. 2010). Their diet is composed of shore crabs, shrimps, polychaetes, gobies, and a variety of other organisms (Hop et al. 1992) often abundant in eelgrass and macroalgae substrates (Lekve et al. 1999; Fredriksen et al. 2009). Studies on other coastal cod populations confirm the importance of vegetated shallow habitats for coastal cod (Gotceitas et al. 1997; Jackson et al. 2001; Persson et al. 2012). The exclusion of cod from such shallow habitats may hypothetically lead to decreases in fish growth during warm summer months. Such effects on fish growth are expected to be greater among large specimens given their stronger responses to increased sea surface temperatures. We hypothesize that in a long term, this may have evolutionary impacts on coastal cod populations, favoring small-sized individuals.

Our study identifies behavioral responses to temperature and the likely ecological consequences of physiological limitation in cod and thereby adds field evidence which complements laboratory-based mechanistic studies (see Metcalfe et al. 2012). Ocean temperature has increased significantly along the Norwegian coast during the last decades (Albretsen et al. 2012), and there are clear signs that cod in this region are already experiencing reduced growth during warm summers (Gjosaeter and Danielssen 2011; Rogers et al. 2011). Further increases in ocean temperature are expected in the near future, with rises between 2 and 4°C being predicted by the end of the century in the Skagerrak and shallow southern North Sea (Ådlandsvik 2008; Dye et al. 2013). In coastal Skagerrak, the cod forms a network of local populations with limited connectivity (Knutsen et al. 2003; Ciannelli et al. 2010) and with an overall decline in abundance in recent decades (Olsen et al. 2009). Results from this study suggest that future and ongoing rises in sea temperature may increasingly exclude cod in this region from shallow feeding habitats during warm summer periods. We hypothesize that exclusion from those areas will affect fish growth and condition during summer and may become detrimental for local populations of the species in Skagerrak and other areas of their southern range of distribution. The Atlantic cod is an ecological keystone species, interacting trophically with numerous other species (Worm and Myers 2003; Frank et al. 2005). The species also holds a major economical and cultural importance. We therefore expect that further reduction or extirpation of cod stocks will have impacts on the biodiversity and stability of the ecosystem, as well as on the economies and livelihoods that depend on them (Schindler et al. 2010).

## Supporting Information

**Figure S1**. Detection range of the acoustic receivers. **Figure S2**. Number of centre-of-activity locations as a function of time. **Tables S1–S10**. Linear mixed-effects model selection tables.
