# Supplementary material for: Behavioral responses of Atlantic cod to sea temperature changes
Source: Ecol Evol. 2015 Apr 17;5(10):2070–83. doi: 10.1002/ece3.1496 (PMC4449760; doi:10.1002/ece3.1496)
Supplement: Supplementary file 1 [file ece30005-2070-sd1.doc]

**Supplementary Figures**

Figure S1. Mean percentage (± standard deviation) of detections recorded as a function of the distance between the test tag and the acoustic receiver.

Figure S2. Mean number (± standard error) of unique center-of-activity locations as a function of the time interval used in the calculation.


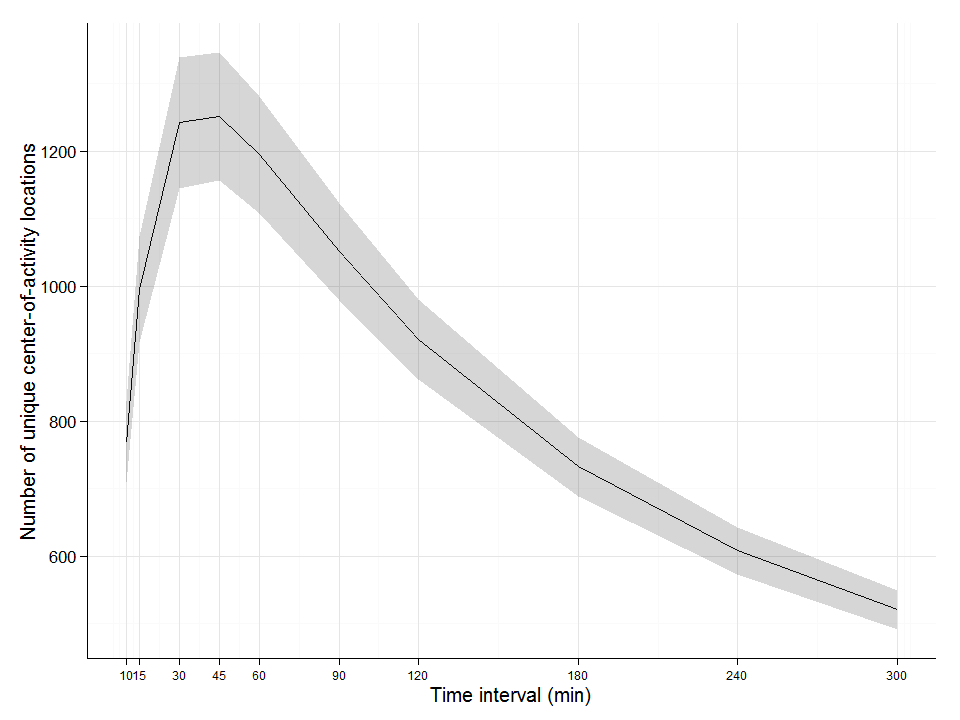


**Supplementary Tables**

Table S1. Model selection for the summer average depth during the day, based on the Akaike’s information criteria (AIC) and Akaike weights (*w_i_*). The 95% confidence set of models (cumulative weights > 0.95) is presented in bold. AIC differences (Δ*_i_*) and cumulative weights (cum *w_i_*) are also shown.

Abbreviations: T1m, temperature at 1 m depth, i.e. surface temperature (°C); T19m, temperature at 19 m depth, i.e. bottom temperature (°C); DifT, difference between surface and bottom temperatures (°C); Up, upwelling (2 categories: 1=presence, 2=absence); Prec, precipitation (mm), Len, fish body size (cm); jul, julian day, starting in January 1^st^ of the tagging year; year, year (4 levels: 2008, 2009, 2011, 2012); dnc, did not converge. Random effects and temporal autocorrelation terms follow the R-function *lme* (library *nlme*) syntax.

Table S2. Model selection for summer average depth at night. See Table S1 for abbreviations.

Table S3. Model selection for summer diel vertical migration. See Table S1 for abbreviations.

* This model did not converge during model selection of fixed effects. The next best random structure was therefore used.

Table S4. Model selection for summer activity level. See Table S1 for abbreviations.

* This model did not converge during model selection of fixed effects. The next best random structure was therefore used.

Table S5. Model selection for average distance moved in summer. See Table S1 for abbreviations.

* This model did not converge during model selection of fixed effects. The next best random structure was therefore used.

Table S6. Model selection for winter average depth at day. See Table S1 for abbreviations.

* This model did not converge during model selection of fixed effects. The next best random structure was therefore used.

Table S7. Model selection for winter average depth at night. See Table S1 for abbreviations.

Table S8. Model selection for winter diel vertical migration. See Table S1 for abbreviations.

* This model did not converge during model selection of fixed effects. The next best random structure was therefore used.

Table S9. Model selection for winter activity level. See Table S1 for abbreviations.

* This model did not converge during model selection of fixed effects. The next best random structure was therefore used.

Table S10. Model selection for average distance moved in winter. See Table S1 for abbreviations.

* This model did not converge during model selection of fixed effects. The next best random structure was therefore used.
